# Differences in Faecal Nutritional Components in Three Species of Saharan Gazelles on Standard Diets in Relation to Species, Age and Sex

**DOI:** 10.3390/ani13213408

**Published:** 2023-11-02

**Authors:** Stipan Čupić, Jorge Cassinello, Tomáš Kušta, Francisco Ceacero

**Affiliations:** 1 Department of Game Management and Wildlife Biology, Faculty of Forestry and Wood Sciences, Czech University of Life Sciences, 16500 Prague, Czech Republic; cupic@fld.czu.cz (S.Č.); kusta@fld.czu.cz (T.K.); 2 Experimental Station of Arid Zones, Spanish National Research Council (EEZA-CSIC), 04120 Almeria, Spain; jorge.cassinello@eeza.csic.es; 3 Department of Animal Science and Food Processing, Faculty of Tropical AgriSciences, Czech University of Life Sciences, 16500 Prague, Czech Republic

**Keywords:** body size, digestive efficiency, feeding ecology, fibre, nitrogen

## Abstract

**Simple Summary:**

The study examines how different factors influence the nutritional content of faeces from three gazelle species, with particular interest in the inter-specific factor. Through the contents of nitrogen and fibre, faeces can tell us about their digestive process. The research focuses on 193 captive individuals of three gazelle species and applied Near InfraRed Spectroscopy technology. The results show that different species have varying faecal nutrient levels. Cuvier’s gazelle had lower nitrogen content, suggesting less efficient digestion than other gazelles. Factors like sex and age also played a role, but their effects were not the same for all species. Fibre content, related to diet quality, remained consistent. This study shows that factors affecting faecal nutrients are species-specific.

**Abstract:**

Various environmental, individual, and species-specific factors may affect digestive efficiency in wild ruminants. The study of faecal nutritional components is a commonly used technique to understand these effects, assuming that faecal nitrogen and fibre contents reflect the diet’s nutritional quality and digestibility. Recent studies have highlighted the relatively high influence of factors like sex, age, weight or body condition on digestive efficiency. This manuscript is focused on the inter-specific variability in faecal nutritional components under the same feeding regime, using three captive populations of closely related gazelles as model species. Faecal samples from 193 individuals were analysed through Near InfraRed Spectroscopy. Species, sex and age influence on faecal nitrogen and fibres (ADF and NDF) were investigated. We found inter-specific differences in the faecal content of the three studied nutritional components. Cuvier’s gazelle showed lower faecal nitrogen content, suggesting lower digestive efficiency than dorcas and dama gazelles. Sex and age also had a moderate effect, especially in faecal nitrogen, but these effects were not constant across the three studied species. On the contrary, faecal fibres were highly constant (i.e., dependent on diet quality). These results confirm that individual factors affecting faecal nutritional components are also species-specific.

## 1. Introduction

Animal feeding ecology is a complex field due to the numerous factors affecting it and the countless interactions among them. These can be divided into environmental (habitat-specific), individual (animal-specific) and species-specific (morpho-physiological) factors [1]. Various techniques are used to understand the feeding ecology of wild species. Among them, the study of faecal nutritional components has been an essential approach to studying the nutritional quality of animal diets for decades, herbivorous mammalian species in particular [2], under the assumption that faecal nitrogen (fN) and fibre (acid detergent fibre (fADF), and neutral detergent fibre (fNDF)) contents reflect dietary ones [3,4], and thus food quality can be estimated. Even if this view is widely accepted [2,5], other studies have suggested that fN measures feed digestibility [6]. Nevertheless, under controlled settings with fixed and equal diets, individual and specific differences in food digestibility arise, informing about digestive efficiency [1]. For samples collected in the wild, researchers often have little or no information about the individual and the feed [7] and, by extension, about the variability related to environmental, individual or species-specific factors as described above, making it challenging to reach sound conclusions [8].

**Figure 1:**
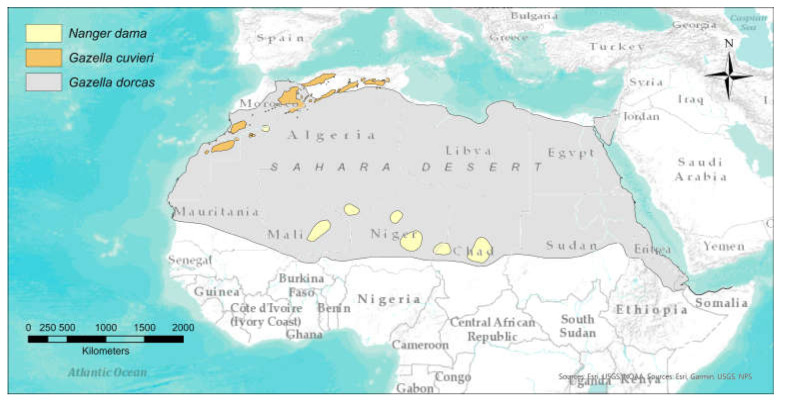
Distribution map of the studied species: *Gazella cuvieri*, *Nanger dama* and *Gazella dorcas* (source: IUCN SSC Antelope Specialist Group [16,17,18], respectively).

In a previous study on captive red deer [1], we demonstrated how environmental and individual factors strongly affect fN, fADF and fNDF under a controlled feeding regime. In this manuscript, we focus on inter-specific variability in faecal nutritional components under a common feeding regime, using three captive populations of gazelles from the Sahel–Saharan region as model species: dama gazelle (*Nanger dama*), Cuvier’s gazelle (*Gazella cuvieri*), and dorcas gazelle (*Gazella dorcas*). Even if closely related, these three species inhabit ecologically distinct habitats (Figure 1), use different food sources, and experience different nutritional demands and challenges due to their different body size and life history traits [9,10,11]. Thus, predicting species-specific strategies in their digestive function and efficiency is reasonable. For example, large body size ungulates may have lower relative energy requirements due to their increased gastrointestinal tract capacity and longer ingesta passage rates [12] or as an adaptation to the feed quality and availability under different ecological conditions [13]; on the contrary, it has been suggested that forage quality plays an especially critical role in the nutritional regulation of small-herbivore species [14] (but see [15]). The reproductive effort is another factor affecting faecal nutritional components at the individual level [1].

Dama gazelle selects a mixed diet based on grazing herbaceous plants and browsing the foliage of woody species in close association with acacia woodlands [19,20,21]. It is the largest of the three studied species. Cuvier’s gazelle favours grasses, young leaves of leguminous plants, perennials and plants associated with maquis [22,23,24]. Among the three studied species, it is the only one that may deliver twins [25]. Moreover, it is the only diurnal one, and thus, may have increased water requirements than the other two species. Dorcas gazelle is distributed along a wider variety of habitats across the region, and their needs for food and water vary significantly across that range. It can survive in areas with no surface water throughout the year. They prefer habitats with trees and shrubs, browsing on acacia groves [26,27,28,29].

NIRS technology has become a widely used method that allows for the rapid, low-cost analysis of the nutritional content of large amounts of samples and is already commonly used for measuring food quality through faecal indices in ungulates [30]. The three study species are threatened in the wild. Thus, this study and the validation of the technique in captivity can lay the foundations for further studies on these species’ feeding and nutritional ecology in their areas of origin. Considering all the previously stated differences between the three species described, we aimed to study inter-specific differences in digestive efficiency under the same feeding regime by analysing faecal nutritional components after controlling for individual factors like sex and age.

## 2. Materials and Methods

### 2.1. Data Collection and Processing

The study was carried out in May 2017 during the yearly handling of the animals for regular health control at “La Hoya” Experimental Farm (FEH) of the Experimental Station of Arid Zones (EEZA-CSIC) in Almería, Spain. One hundred and ninety-three healthy animals were studied, out of which 100 were dama gazelles (37 males and 63 females), 21 Cuvier’s gazelles (7 males and 14 females) and 72 dorcas gazelles (38 males and 34 females). The animals used in this research ranged from 1 to 14 years old for dorcas and Cuvier’s, and 1 to 17 for dama gazelle. Pregnant individuals were not considered for this research to avoid causing eventual stress.

All animals were kept in spacious paddocks with bare soil and no pasture provided, minimising soil ingestion and the transmission of nematodes, which can be a confounding factor in nutritional studies [6]. Animals of different species were assigned to separate paddocks and subdivided according to population management needs, from isolated animals to small breeding groups. Animals were fed daily with a combined diet of fresh alfalfa (*Medicago sativa*), wheat and feed pellets for herbivores (composition shown in Table 1). Each feedstuff, water and mineral licks were provided ad libitum to avoid competition and selection [31]. This combination of feedstuff has been successfully used for many years at FEH, ensuring constant protein availability and an adequate source of fibre, which is important for proper gut function. The ratio of provided feedstuffs changed slightly over the year according to seasonal needs. Still, it was constant for the previous month before the samples for this research were collected. No further measuring of leftovers and the exact amount of each feed component in each paddock was possible since the husbandry protocols are designed to minimise contact with the animals to reduce stress.

Handling and sampling procedures carried out at the farm were designed during the routine yearly handling of the animals for veterinary inspection under the expertise and supervision of the veterinarian in charge, who complies with the authorisations established by Spanish regulations on animal welfare. Animals were hand captured by net, immobilised in the ground with covered eyes, identified, and visually inspected as described and advised in the studbook of Cuvier’s gazelle [32]. To reduce contamination, the faecal samples were collected from the rectum while animals were immobilised, just after routine blood sampling. Samples were dried to a constant weight in a hot air dryer at 40 °C for 48 h, ground with a mill to pass through a 1 mm sieve, and thoroughly mixed to achieve maximum homogeneity. The same approach was used for four subsamples of each feedstuff previously described. All the samples were subsequently scanned with the NIRS™ DS 2500 FOSS analyser under the ISIscan^TM^ 4.10 Routine Analysis Software (Foss, Hillerød Denmark), which is a rapid, low-cost, chemical-free, and non-destructive analysis method rapidly developing [7,33]. By this method, the contents of fN, fNDF and fADF were calculated using WinISI 4 Calibration Software (Foss, Hillerød, Denmark) according to a calibration set previously developed from a subset of the main sample set, which was analysed using conventional wet chemistry methods (NEN-ISO 5983-2 for protein; EN-EN-ISO 16472:2006 for NDF; NEN-EN-ISO 13906:2008 for ADF; [34]). For the calibration, we chose 34 samples out of the 193 samples collected (14 from dama gazelle, 12 from dorcas gazelle and 8 from Cuvier’s gazelle), which is representative of the dataset regarding animals’ body weight, age and sex. The wet chemistry confirmed that neither sand nor any other contaminants or dust affected the purity of the samples. Also, the accuracy of the calibration set was strengthened by adding faecal samples from red deer [35], reaching adequate goodness-of-fit indicators for the samples analysed (average GH1 = 0.912; NH1 = 0.168). The nutritional content of feedstuffs was calculated using standard calibration packages (Foss, Hillerød Denmark).

### 2.2. Statistical Analyses

The normality of the continuous variables studied was confirmed through Kolmogorov–Smirnov tests, and the homogeneity of variances was confirmed through Levene´s test. A multivariate general linear model was conducted to understand the effects of Species, Sex and Age on the studied faecal nutritional components: fN, fADF and fNDF. The interactions Species*Sex and Species*Age were also included in the model since the preliminary inspection of the data suggested sex-related differences in at least one species. Analyses were performed using IBM^®^ SPSS^®^ Statistics (version 29.0 for Windows, IBM, USA).

## 3. Results

Species (Wilks’ λ = 0.667; F_6,364_ = 13.592; *p* < 0.001), the interaction Species*Sex (Wilks’ λ = 0.844; F_6,364_ = 5.374; *p* < 0.001), and Age (marginally; Wilks’ λ = 0.962; F_3,182_ = 2.397; *p* = 0.070) showed a significant influence in the studied faecal nutritional components, while Sex (Wilks’ λ = 0.995; F_3,182_ = 0.307; *p* = 0.820) and the interaction Species*Age (Wilks’ λ = 0.965; F_6,364_ = 1.091; *p* = 0.367) were not.

**Figure 2:**
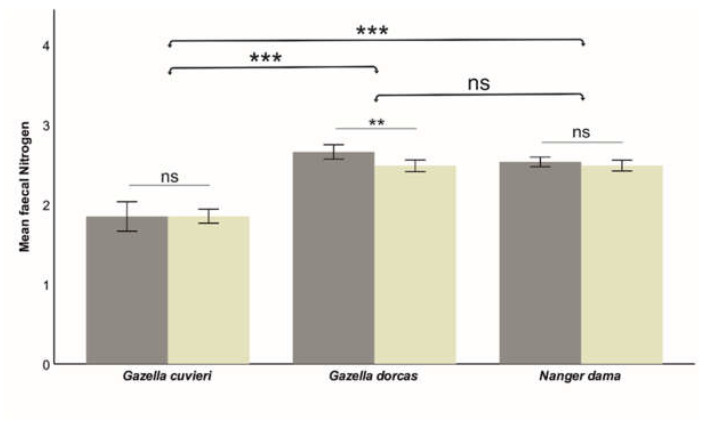
Influence of species and sex (dark bars correspond to females) on the measured faecal nitrogen (% dry matter) was lowest in Cuvier´s gazelle with respect to the other two studied species. Sex differences were found only for dorcas gazelle. Means ± SD (bars) are shown. Significance is indicated at *p* < 0.001 (***) and *p* < 0.010 (**) levels; ns indicates a lack of significance.

**Figure 3:**
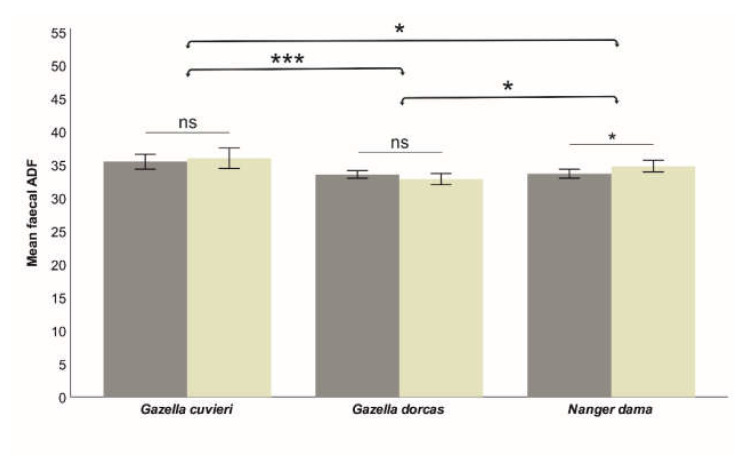
Influence of species and sex (dark bars correspond to females) on the measured faecal ADF (% dry matter), which was significantly different for each species. Sex differences were found only in dama gazelle. Means ± SD (bars) are shown. Significance is indicated at *p* < 0.001 (***) and *p* < 0.050 (*) levels; ns indicates a lack of significance.

**Figure 4:**
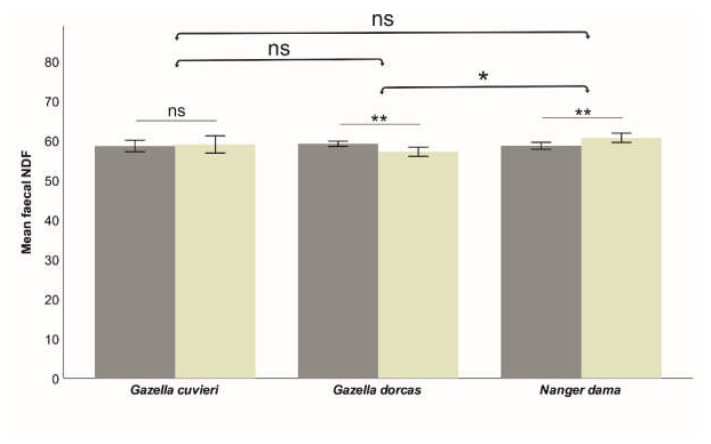
Influence of species and sex (dark bars correspond to females) on the measured faecal NDF (% dry matter), which was only significantly different between dorcas and dama gazelles. Sex differences were found in the same two species but not in Cuvier´s gazelle. Means ± SD (bars) are shown. Significance is indicated at *p* < 0.010 (**) and *p* < 0.050 (*) levels; ns indicates a lack of significance.

The model was quite robust for fN (R^2^ = 0.493), which was affected by Species, Age and the interaction Species*Sex. However, the models were relatively weak for fADF (R^2^ = 0.125, significantly affected only by Species) and fNDF (R^2^ = 0.111, significantly affected by Species—marginally—and the interaction Species*Sex). That indicates that individual factors moderately influence faecal nitrogen, while faecal fibres are weakly influenced by individual characteristics but strongly dependent on diet quality. The effects of these factors on each of the studied faecal nutritional components are shown in Table 2. Species significantly affected fN (lower in Cuvier’s gazelle than in dorcas and dama gazelle; Figure 2). Species also affected the faecal fibres (fADF and fNDF; Figure 3 and Figure 4), although the differences were much smaller (6.9% for fADN and 2.2% for fNDF; differences between the largest and smaller average values across the three studied species) compared to fN (27.9%). The effect of Sex was different across species. In dama gazelle, faecal fibres were lower in females (t = −2.010, *p* = 0.046 for ADF; t = −2.771, *p* = 0.006 for NDF). In dorcas gazelle, fN (t = 3.380, *p* = 0.001) and fNDF (t = 2.528, *p* = 0.012) were higher in females. In Cuvier’s gazelle, no sex-related differences in faecal nutritional components were found. Faecal nitrogen significantly increased with Age (t = 2.921, *p* = 0.004), while faecal fibres were not affected.

## 4. Discussion

In a controlled research setting with three closely related species (dama, Cuvier’s and Dorcas gazelles) under the same feeding regime, we found inter-specific differences in the faecal content of three studied nutritional components: N, ADF and NDF. Cuvier’s gazelle showed a significantly lower amount of fN than the other species, suggesting lower rumen microbial activity and thus lower digestive efficiency. Individual factors like sex and age also moderately influenced the faecal content of nutritional components, especially fN, but these effects were not constant across the three studied species. On the contrary, faecal fibres were highly constant (i.e., highly dependent on diet quality). Since the diet was the same for all the studied animals, the results show differences in digestive efficiency but not diet selection nor digestibility.

Recent intra-specific research [1] found that individual factors, like sex, age, reproductive status, body mass, body condition, season (linked to different nutritional requirements for each sex) and presence/absence of natural pasture, significantly affected faecal nutritional components in a similar experimental setting with captive red deer. In that study, separate analyses were conducted for males and females due to the large sexual dimorphism linked to different nutritional requirements in the species at different periods of the yearly cycle. Still, differences in faecal nutritional components between sexes could be confirmed since these were explained by different factors. In gazelles, sex was not an important factor per se. However, it was significant in interactions within each species: no differences between sexes of Cuvier’s gazelle; higher fN and fNDF in females of dorcas gazelle; and higher fADN and fNDF in males of dama gazelle. In general, these significant differences were low compared with our previous study on red deer, which may be related to the smaller sexual dimorphism in body size among gazelles compared with deer [36,37]. Surprisingly, no sex differences were found in Cuvier’s gazelle, which is a species with certain sexual dimorphism and greatest reproductive outputs (twins are common), so greater efficiency in females of this species could be expected even if we did not use pregnant or lactating females in this study. Thus, further studies are necessary to fully understand sexual differences in digestive efficiency in gazelles and other ungulates, and how it is linked to sexual dimorphism.

Age was the other individual factor studied. Age had a significantly positive effect on fN but not on faecal fibres. This result is again different to the one observed in red deer [1]. In that species, fN decreased with age (i.e., lower efficiency), and changes in faecal fibres were observed. In general, the protein requirements in ruminants decrease with age [38], which seems to be the easiest explanation for the fN increase observed in this study. It may be argued that these differences may be due to the presence of tannins and other plant secondary compounds in the feed, which may decrease protein digestibility and increase its excretion [39,40]. However, this is unlikely in our setting since only common feedstuffs for livestock with low content of plant secondary compounds were used.

This study aimed to investigate species-specific differences in faecal nutritional components in related species with different ecological characteristics under the same feeding regime. This was clearly observed for fN, which indicates different digestive efficiency among the studied species but not for faecal fibres, confirming that they are weakly affected by specific or individual factors (low R^2^ in the models) but strongly dependent on diet quality, which was the same for the three species. Thus, we will focus on the results obtained for fN. These were similar for dorcas and dama gazelles but lower for Cuvier’s, suggesting a lower ruminal activity and digestive efficiency in this species. This is further supported by the greater fNDF observed compared to the other species. The previously commented greater reproductive performance of Cuvier’s gazelles may explain this result. Indeed, the lowest fN would have been expected in dama gazelle. Digestive efficiency is directly related to retention time, a species-specific parameter determined by body mass [41]. While the three species are considered browsers with a certain flexibility in diet selection, the dama gazelle shows a higher degree of grazing [42], which may also explain the different digestive efficiency observed among species. Finally, water requirements may be another ecological factor explaining the results. Among the three species, Cuvier’s gazelle has greater water requirements. In the wild, since most of these requirements are satisfied by the water content of plants, it may affect the natural diet selection. Thus, the species may prioritise the water over the protein content of the plants, which may explain the decreased digestive efficiency that the results suggest.

## 5. Conclusions

These results confirm our previous finding on individual factors affecting faecal nutritional components but also show that these individual factors may work differently for different ungulate species, even if taxonomically closely related. Moreover, the results show that while faecal fibres are a reliable indicator of diet quality across species, faecal nitrogen is not because of species-specific differences in digestive efficiency. Thus, comparative studies based on faecal nutritional components for different species sharing distribution may be considered carefully and may benefit from preliminary studies with captive individuals and controlled diets. That seems the only reasonable way to interpret samples collected in the wild adequately.

## Figures and Tables

**Table 1:** Nutritional content of the feedstuff provided to the study animals in percentage of dry matter.

	Protein (%)	ADF (%)	NDF (%)
**Fresh alfalfa**	19.7	45.2	51.5
**Wheat**	26.4	20.3	28.9
**Pellets**	17.9	16.3	34.6
**Dry silage**	9.5	47.3	71.9

**Table 2:** Influence of the selected factors on each of the studied faecal nutritional components. Significance is indicated at *p* < 0.001 (***), *p* < 0.010 (**), *p* < 0.050 (*) and *p* < 0.100 (†) levels.

	fN	fADF	fNDF
R^2^	0.493	0.125	0.111
Intercept	F = 4669 ***	F = 10344 ***	F = 17067 ***
Species	F = 34.530 ***	F = 6.606 **	F = 2.637 †
Sex	F = 0.769	F = 0.086	F = 0.001
Age	F = 6.355 *	F = 0.681	F = 0.360
Species*Sex	F = 3.459 *	F = 2.200	F = 6.613 **
Species*Age	F = 0.398	F = 0.622	F = 0.064

## Data Availability

The data used to support the findings of this study are available from the corresponding author upon request.
